# Comparison of supine–prone percutaneous nephrolithotomy methods in the treatment of kidney stones in pediatric patients: prospective randomized study

**DOI:** 10.1007/s00240-024-01543-w

**Published:** 2024-05-02

**Authors:** Recep Eryılmaz, Kasım Ertas, Rahmi Aslan, Mehmet Sevim, Muhammed Fatih Keles, Kerem Taken

**Affiliations:** 1 Yuzuncu Yil University Faculty of Medicine, Van, Turkey; 2 Siirt Education and Training Hospital, Siirt, Turkey

**Keywords:** Pediatric, PCNL, Supine, Prone

## Abstract

Mini-PCNL is one of the most effective surgical methods in the treatment of kidney stones in pediatric patients. In this study, we aimed to compare PCNL in the supine–prone position in pediatric patients (especially operation time, postop complications, hospital stay and stone-free rates).We conducted our study in a randomized and prospective manner. Patients with lower pole stones larger than 1 cm, stones larger than 1.5 cm in the pelvis, upper pole, midpole or multiple locations, and patients who did not respond to ESWL or whose family that preferred mini-PCNL to be the primary treatment were included in the study. Patients with any previous kidney stone surgery, patients with coagulation disorders and patients with retrorenal colon were excluded from the study. Between 2021 and 2023, a total of 144 patients underwent PCNL. 68 of these patients had supine PCNL and 76 prone PCNL. Postoperative Clavien grade1 complication occurred in a total of 7 patients in the prone position; Clavien grade1 complication occurred in 1 patient in the supine position. The mean operation time for prone PCNL was 119.88 ± 28.32 min, and the mean operative time for supine PCNL was 98.12 ± 14.97 the mean hospitalization time in prone PCNL was 3.56 ± 1.12 days, and 3.00 ± 0.85 days in supine PCNL. In conclusion, supine PCNL is a safe and effective method in the treatment of pediatric kidney stones and postoperative complications were observed to be less; the operation time and hospital stay were shorter in supine PCNL.

## Introduction

Percutaneous nephrolithotomy (PCNL) was first performed in the pediatric age group by Helal et al. [1]. Later, Jackman et al. performed the classical PCNL using a smaller Amplatz sheath. Over time, a miniaturization developed in the endoscopic instruments used with technological developments and mini-PCNL replaced the standard PCNL in children. Complication rates decreased with mini-PCNL [2, 3].

Today, the treatment for kidney stones larger than 2 cm is PCNL. This is the recommendation of both American urology and European urology associations, prone PCNL has been used safely in kidney stones for years with minimal complications. Today, PCNL in the prone position is performed more frequently, but surgeons continued to search for different techniques and Valdivia et al. described the supine position PCNL operation for the first time in 1987, approximately 11 years after prone PCNL and this surgical technique is becoming more and more widespread thanks to its advantages such as shortening the surgical time as a result of not changing the position and simultaneously being able to intervene in the patient from the urethra endoscopically, and most importantly, ease of anesthesia control and monitoring [4].

Giusti et al., in their study, reported that supine PCNL is a promising method and that it will become the standard PCNL method in the future, thanks to the advantages it provides [5].

In this study, we aimed to prospectively compare PCNL in supine and prone positions in our pediatric patients with kidney stones (especially operation time, postop complications, hospital stay and stone-free rates and so on).

## Materials and methods

### Study design

After the ethical approval of the study, pediatric patients (< 16 years old) who underwent prone and supine Mini-PCNL for kidney stones in our clinic between April 2021 and August 2023 were included in the study. We conducted our study in a randomized and prospective manner. Patients with lower pole stones larger than 1 cm, stones larger than 1.5 cm in the pelvis, upper pole, midpole or multiple locations and patients who did not respond to ESWL or whose family that preferred mini-PCNL to be the primary treatment were included in the study. Patients with any previous kidney stone surgery, patients with coagulation disorders and patients with retrorenal colon were excluded from the study. İn summery, most of the cases were primary cases, only some cases consisted of patients who had passed ESWL and whose stones were not broken. Since the patients were children, they were operated after obtaining consent from their parents.

Complete urinalysis, urine culture, direct urinary system radiography, ultrasonography (USG), and non-contrast computed tomography (CT) were performed from all patients included in the study. Patients with growth in urine culture were operated after being treated with appropriate antibiotic therapy.

The average stone load was determined by multiplying the longest dimension of the stones and the length of the other side by the right angle to it. More than one stone was measured one by one and total stone size was obtained. Holmium laser was used for fractionation of stones.

## Surgical technique

### Supine PCNL

After prophylactic administration of 1 × 1000 mg ceftriaxone intravenously, the patient was taken to the operating table and general anesthesia was administered to all patients. After anesthesia, a 4f ureteral catheter was inserted by ureterorenoscopy on the side to be operated on, in the lithotomy position, and then a urethral catheter was inserted. The ureteral catheter was fixed to the urethral catheter. On the side of the patient to be operated on, a linear line was drawn from the patient’s posterior axillary line to the superior anterior iliac bone with a surgical pencil, then a line was drawn from under the 12th rib to the patient’s back with a surgical pencil, and finally, a line was drawn from the patient’s superior anterior iliac bone region to the back with a surgical pencil. The kidney was accessed from the area between these three lines. Then the patients were placed in the Galdakao Modified Supine Valdivia position. As described in this position, the ipsilateral lower extremity of the patient was brought into extension, and the contralateral extremity was abducted and flexed.

A silicone pad was left on the underside of the area to be accessed and this area was lifted approximately 20 degrees. The arm on the same side was fixed on the rib cage, crossing the rib cage and leaving a pillow under it. By performing retrograde pyelography, the renal calyx to be accessed was determined and access was provided by entering the kidney from the region we previously determined. Dilatation was performed with the help of Amplatz dilators (Microvasive/Boston Scientific, Natick, MA) up to 14 fr and entered into the collecting system with a 14 fr nephroscope (Karl Storz).The stones were broken by holmium laser (wolf: 8–10 Hz, 1500–2000 J). The collecting system and ureteral passage were checked with antegrade pyelography before the surgery was terminated. The procedure was terminated by placing a 12 Fr percutaneous Malecot nephrostomy in the patients.

### Prone PCNL

Before the patient was taken to the operating table, 1000 mg of ceftriaxone was administered. Afterwards, the patient was given general anesthesia and the patient was placed in the lithotomy position and cystoscopy was performed. Preferably, open-ended 4 Fr ureteral catheter was placed in the ureter on the planned side of the operation. After the ureteral catheter was left in the pelvis, the urethral catheter was inserted and the ureteral catheter was fixed to the catheter. Then, the patient was carefully placed in the prone position. When the patient was placed in the prone position, silicone pillows were placed on the chest area, both flank areas, and under the feet, especially to prevent lung compression The area to be accessed and the genital areas of all patients were painted with antiseptics, a sterile drape was provided and the tip of the 4 fr ureteral catheter sent through the urethra was sterilized. Then the contrast agent given in the 4 fr ureteral catheter, retrograde pyelography was provided and the appropriate calyx was determined accordingly and the kidney was accessed. After 14 fr dilatation, 14 fr Amplatz was placed and the kidney was accessed with a 14 fr nephroscope. Holmium laser was used for stone fragmentation (wolf: 8–10 Hz, 1500–2000 J). The collecting system and ureteral passage were checked with antegrade pyelography before the surgery was terminated. The procedure was terminated by placing a 12 fr percutaneous Malecot nephrostomy in the patients.

### Statistical Analysis Report

In calculating the sample size of this study, which was conducted to compare the supine–prone percutaneous nephrolithotomy methods in the treatment of kidney stones in pediatric patients, power was determined by taking at least 80% and Type-1 error of 5% for each variable. Kolmogorov–Smirnov and skewness–kurtosis tests were used to check whether the continuous measurements in the study were normally distributed, and because the measurements were normally distributed, parametric tests were applied. Descriptive statistics for the variables in the study; expressed as mean, standard deviation, number (*n*) and percentage (%). The “Independent *T* test” was calculated in the comparison of the measurements according to the “groups”. Chi-square test was calculated to determine the relationships between categorical variables. Statistical significance level (a) was taken as 5% in the calculations and SPSS (IBM SPSS for Windows, ver.26) statistical package program was used for analysis. *p* < 0.05 was found to be statistically significant.

## Results

Between 2021 and 2023, a total of 144 patients underwent PCNL. 68 of these patients had supine PCNL and 76 prone PCNL. In the prone PCNL group, 40 were male and 36 were female, in the supine PCNL group, 38 were male and 30 were female. The mean age of the patients was 6.34 ± 3.6 in the prone PCNL group and 7.47 ± 4.13 in the supine PCNL group. Mean stone size was 16.27 ± 4.15 mm in prone PCNL, 17.03 ± 4.58 mm in supine PCNL, mean hospitalization time in prone PCNL was 3.56 ± 1.12 days, and 3.00 ± 0.85 days in supine PCNL. The mean operation time for prone PCNL was 119.88 ± 28.32 min, and the mean operative time for supine PCNL was 98.12 ± 14.97. Of the stones in the prone position, 4 was located in the upper pole, 24 in the middle pole, 9 in the lower pole, and 39 in the renal pelvis. Of the stones in the supine position, 5 were located in upper pole, 20 were located in the middle pole, 6 in the lower pole and 37 in the renal pelvis. 39 of the stones in the prone position were located in the right kidney, 37 in the left kidney. 36 of the stones in the supine position were located in the right kidney and 32 in the left kidney. The mean preop hemoglobin value in the prone position was 14.37 ± 1.03 and the postoperative hemoglobin value was 12.90 ± 1.15. The mean preop hemoglobin value in the supine position was 13.39 ± 1.50 and the postoperative hemoglobin value was 12.46 ± 1.48. The preop creatinine value of the patients who underwent PCNL in the prone position was 0.52 ± 0.11 and the postoperative creatinine value was 0.54 ± 0.10. The preop creatinine value of the patients who underwent PCNL in the supine position was 0.56 ± 0.13 and the postoperative creatinine value was 0.57 ± was 0.11. Residual stones were seen in only 6 of 76 patients in the prone position. In the supine position, residual stones were seen in 5 of 68 patients. Ureteral catheters were placed in 61 of the panels made in the prone position and DJ stent was placed in 15 of them. Ureteral catheter was inserted in 56 patients and DJ stent was inserted in 12 patients for supine PCNL. 3 of 76 patients had blood transfusion in the prone position. 1of 68 patients had blood transfusion in the supine position. Of the stones in which PCNL was performed in the prone position, 69 were opaque and 7 were non-opaque. 63 of the stones in the supine position were opaque and 5 were non-opaque. We performed a single access for all patients in the supine position. In 3 of the patients who underwent PCNL in the prone position, a second access was made from the upper pole because their stones in the upper pole could not be reached from the access in the lower pole.

Complications according to postoperative Clavien–Dindo; Clavien grade1 complication occurred in a total of 7 patients in the prone position, Clavien grade1 complication occurred in 1 patient in the supine position. Clavien grade2 complications occurred in 2 patients in the prone position and 1 patient in the supine position. Clavien grade3 complications occurred in 3 patients in the prone position, and 2 patients in the supine position. No Clavien grade 4 complications were observed in either group (Tables 1 and 2).

**Table 1:** Demographic and preoperative data

	Prone PCNL (*n* = 76)	Supine PCNL (*n* = 68)	*p
	Mean ± SD or *N* (%)	Mean ± SD or *N* (%)	
Age (year)	6.34 ± 3.60	7.4 ± 4.13	0.105
Stone size (mm)	16.27 ± 4.15	17.03 ± 4.58	0.301
Gender			
Male	40 (52.63)	38 (55.88)	0.696
Female	36 (47.36)	30 (44.11)	
Side right/left			
Right	39 (51.31)	36 (52.94)	0.845
Left	37 (48.68)	32 (47.05)	
Stone placement			
Upper pole	4 (5.26)	5 (7.35)	0.877
Middle pole	24 (31.57)	20 (29.41)	
Lower pole	9 (11.84)	6 (8.82)	
Pelvis	39 (51.31)	37 (54.41)	
Opacity			
Yes	69 (90.78)	63 (92.64)	0.687
No	7 (9.21)	5 (7.35)	

*SD* standard deviation

*Significance levels according to chi-square test or *T* test results

**Table 2:** Intraoperative and postoperative data

	Prone PCNL (*n* = 76)	Supine PCNL (*n* = 68)	*p
	Mean ± SD or *N* (%)	Mean ± SD or *N* (%)	
Hospital duration (day)	3.56 ± 1.12	3.00 ± 0.85	**0.019**
Operation (minute)	119.88 ± 28.32	98.21 ± 14.97	**0.001**
Residual stone			
Yes	6 (7.89)	5 (7.35)	0.903
No	70 (92.1)	63 (92.64)	
Ureter/DJ attachment			
Ureter	61 (80.26)	56 (82.35)	0.748
DJ	15 (19.73)	12 (17.64)	
Transfusion			
Yes	3 (3.94)	1 (1.47)	0.367
No	73 (96.05)	67 (98.52)	
Postoperative complication (the Clavien–Dindo classification)			
Grade 1	7 (9.21)	1 (2.94)	**0.010**
Grade 2	2 (2.63)	1 (1.47)	0.386
Grade 3	3 (3.94)	2 (2.94)	0.519
Grade 4	0 (0.0)	0 (0.0)	–

*SD* standard deviation

*Significance levels according to chi-square test or *T* test results

## Discussion

Although PCNL continues to be performed more frequently in the prone position today, supine PCNL production is increasing. According to the Global PCNL study of the Endourology Society Clinical Research Office, approximately 20% of all PCNLs currently performed constitute supine PCNL. The incidence of retro-renal colon position in the supine and prone positions is 1.9% vs. 10%, respectively. Also, the anesthetic advantages of the supine position are clearly more visible [6, 7].

Supine PCNL certainly offers some advantages over prone PCNL, especially in terms of the anesthetist’s management of the patient. There is better access to the patient and less risk of central and peripheral nerve damage for cardiovascular and pulmonary management in an emergency. Other advantages of the supine position are less vascular, peripheral nerve and cervical spine injuries, tracheal compression, and less ocular damage. On the other hand, since fluid absorption is less in the supine position, it is especially important in pediatric patients, patients with impaired cardiovascular status, and patients at risk of systemic infection due to struvite or non-struvite stones colonized by bacteria [8, 9].

In the supine position with reduced intrarenal pressure, the angle of the renal sheath is typically parallel to the floor or angled downward from the floor. This is the reverse of the orientation of the renal sheath during prone PCNL, where the sheath is angled toward the ceiling (i.e., up from the floor). The angulation of the renal sheath during supine PCNL therefore results in increased renal drainage of fluids and stone fragments. Such continued spontaneous discharge of fluid from the renal pelvis in the supine position could potentially lead to reduced renal pelvis pressures and a theoretically lower infectious risk after PCNL [10]. In patients with supine PCNL, we observed that the stones spontaneously came out with fluid after the stone was fragmented during the operation. Since we performed PCNL in the prone position, we did not see that the stones came out spontaneously in the patients. No significant postoperative infection was observed after the operations performed with both surgical methods.

For kidney stones of pediatric patients, supine PCNL is an effective and safe method. It has advantages such as easy access from the upper pole to the lower pole, low risk of fluid absorption and hypothermia, and easy anesthetic monitoring [6, 11].

İn this study, patients who underwent supine PCNL had a single access from the lower pole of the kidney. With this axess, both the stones in the lower pole were cleaned and the stones in the upper pole were easily reached. Since we could not reach the upper pole stones of 3 of the patients with whom we performed prone PCNL, a second access was made and the upper pole stones were cleaned in this way.

In the study of Desoky et al., supine and prone methods for the treatment of kidney stones in pediatric patients were compared and the operation time was found to be statistically significantly shorter in the supine position, but no significant difference was found in the stone-free rates, postoperative complication rates, postoperative pain and hospital stay [12].

It has been determined that percutaneous nephrolithotomy performed in the supine position requires a shorter operation time, shorter hospital stay and less analgesia requirement compared to percutaneous nephrolithotomy performed in the prone position. Therefore, it is seen that supinPCNL is easier, faster and has less complication rate compared to prone PCNL [13]. In our study, Clavien grade 2 and 3 complications as postoperative complications were similar in both groups, but grade 1 complication was more common in the prone position (*p* < 0.05). Most of these complications were related to position-related pain and fever due to position-related atelectasis.

Therefore, we have seen that PCNL in the supine position is a very comfortable surgical method for the patient. We observed that the operation time is shorter (*p* < 0.05), the patient takes less anesthetic agent (*p* < 0.05) (the operation time is also the time during which the patient receives anesthesia) and hospital stay is shorter (*p* < 0.05) in patients who underwent supine PCNL than the patient who underwent prone PCNL.

As it is known, one of the complications of PCNL is colon perforation. The risk of colon perforation is higher in the prone position than in the supine position [14]. In our study, colonic perforation was not observed in both methods.

In the comparison of supine PCNL in kidney stones performed by Peng Wu and his friends, stone free, bleeding risk rates were found to be similar in both techniques, but the operation time was found to be shorter in the supine position [15].

In our study, no significant difference was observed between the two groups in preoperative and postoperative hemoglobin values, postoperative preoperative creatinine values and stone-free rates.

The small number of patients is one of the limitations of our study. Larger series studies are needed. In studies with larger case numbers, there may be serious differences in postoperative Clavien grade 2–3 complications.

In conclusion, supine PCNL is a safe and effective method in the treatment of pediatric kidney stones. It provides easier access from the lower calyx to the upper calyx. Anesthetic control becomes more comfortable. In addition, performing the percutaneous procedure without changing the patient’s position, less postoperative complications, shortens the operation time and hospital stay statistically significantly compared to the prone position.

## Data Availability

The datasets generated and analyzed during the current study are available from the corresponding author on reasonable request.
